# Supplementary material for: Direct observation of dynamic protein interactions involving human microtubules using solid-state NMR spectroscopy
Source: Nat Commun. 2020 Jan 2;11:18. doi: 10.1038/s41467-019-13876-x (PMC6940360; doi:10.1038/s41467-019-13876-x)
Supplement: Supplementary file 1 — Supplementary Information [file 41467_2019_13876_MOESM1_ESM.pdf]

Supplementary Information

Direct observation of dynamic protein interactions involving human microtubules using solid-state NMR spectroscopy

Luo et al.,

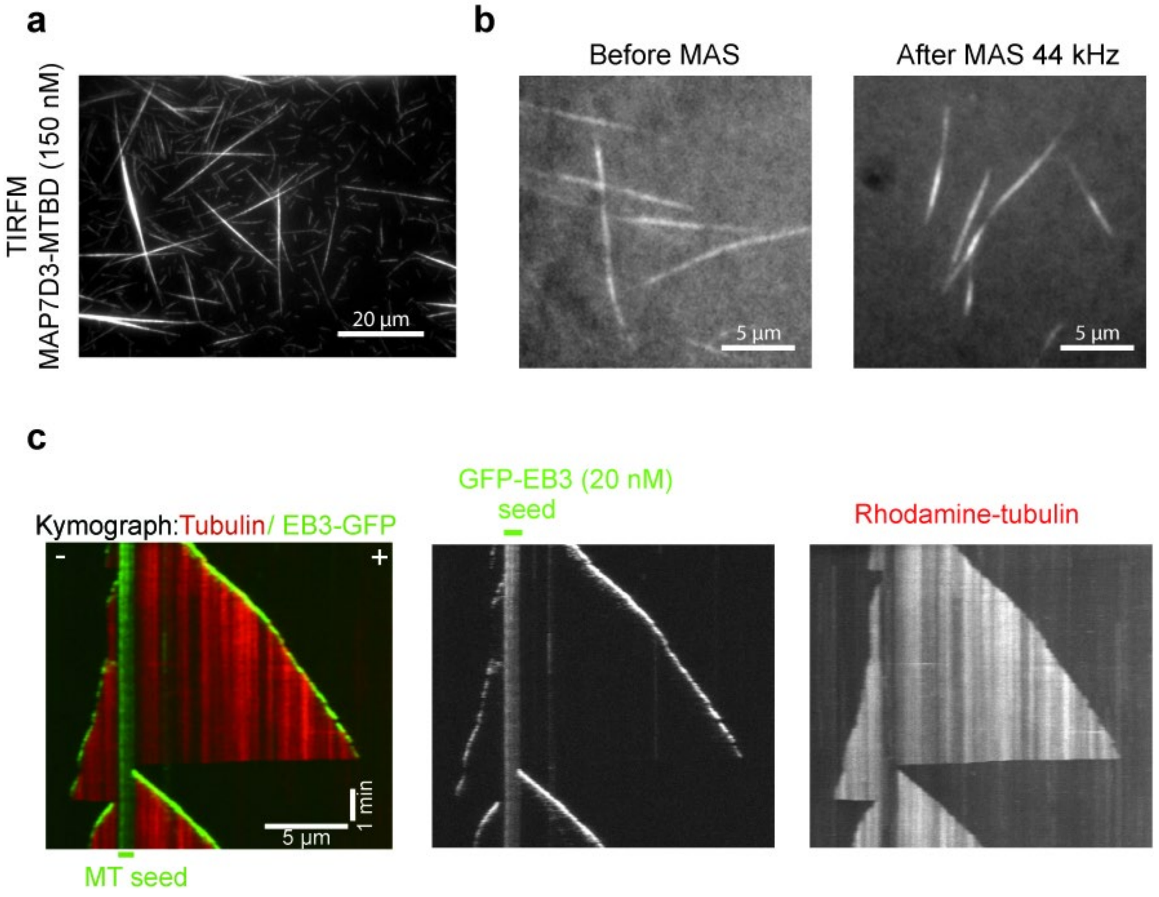

Supplementary Figure 1 | Functional characterization of purified tubulin.

A) MTs were observed by TIRF microscopy using purified mCherry-labeled MAP7D3-MT binding domain (150 nM) as labeling agent. The MT sample was stabilized by 20 µM Taxol. B) Characterization of functionality of HeLa MTs after MAS NMR experiments. Taxol-stabilized MTs were incubated with purified mCherry-labeled MAP7D3-MTBD (150 nM) before or after MAS spinning and observed by TIRF microscopy. C) Kymographs of MT growth at the plus (+) and minus (−) end from a HiLyte-488 seed with 20 nM GFP-EB3 and 3% rhodamine-labeled tubulin together with purified HeLa S3 tubulin. The panel corresponds to the merged kymograph image shown in Figure 1C of the main text.

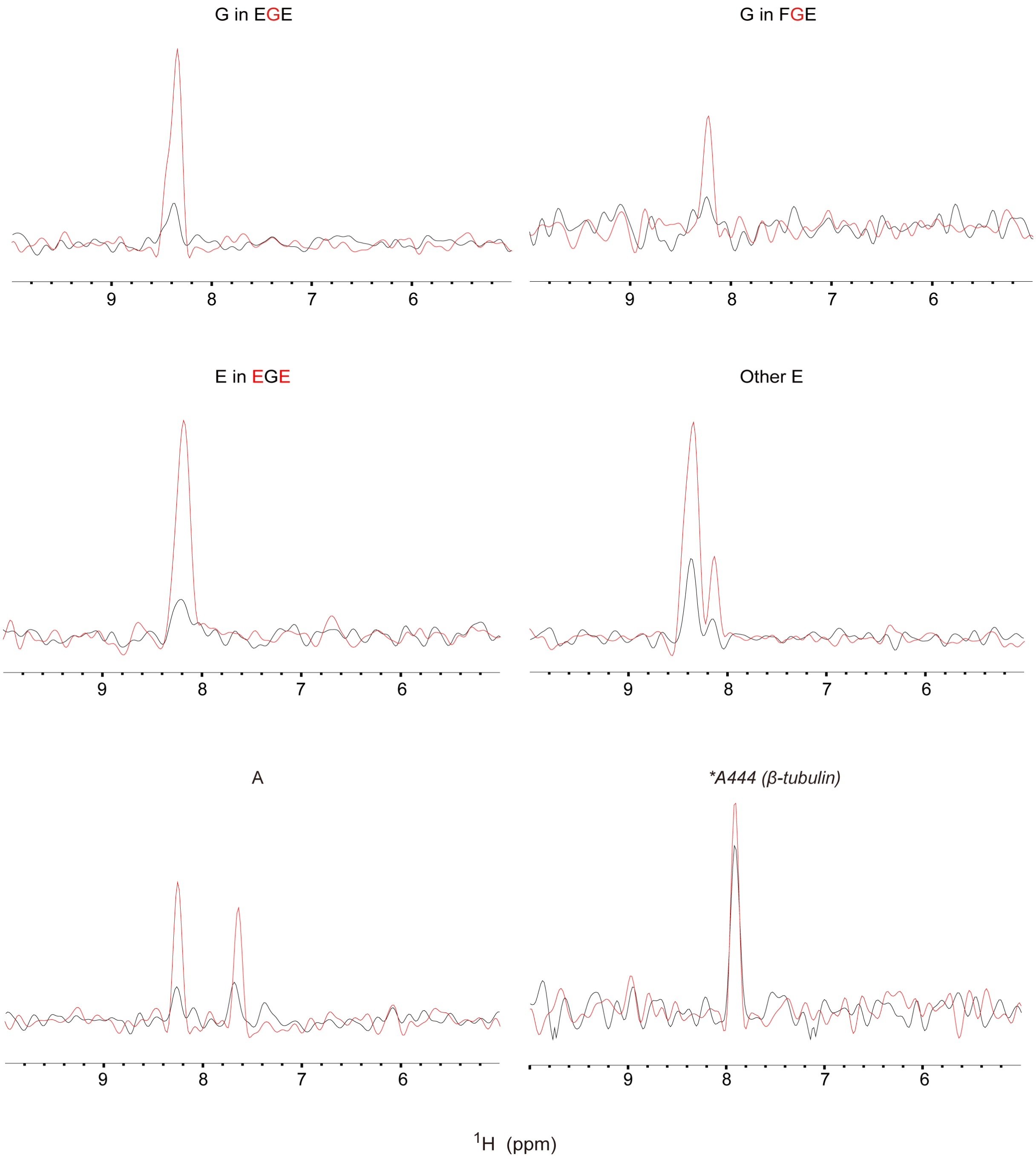

Supplementary Figure 2 | The effect of MAP7 binding on ^{15}N- HSQC data on labeled MTs. state. 1D slices taken from the ^{15}N-HSQC experiments of MTs (red) and MAP7-MTBD in complex with MTs (black). The labels of the peaks are indicated as shown in Figure 5A. NMR intensities are provided as a Source Data file.

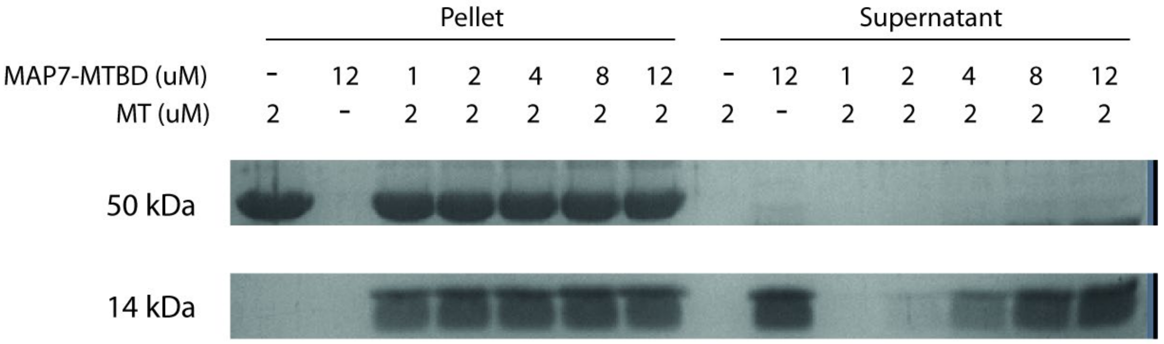

Supplementary Figure 3 | Microtubule pelleting assay with MAP7 analyzed by SDS-PAGE. The MT pelleting assay was performed with different molar ratios of MAP7-MTBD:tubulin as indicated above the SDS-PAGE gel. As a result, a molar ratio of MAP7-MTBD:MTs=2:1 was used for sample preparation for ssNMR studies to allow maximum decoration of MAP7-MTBD on MTs. Uncropped protein gel is provided as a Source Data file.

Supplementary Table 1 | Spectral analysis and results of ^{31}P ssNMR

	δ (ppm)	Integrations
Pα and Pα’	-10.08 and -11.6	0.6589 and 0.639
Pγ and Pβ’	-7.18 and -8.15	1.0000 and 1.1272
Pβ	-19.9	0.5762
Co-purified lipids	-2.7	2.1469

Supplementary Table 2 | Primer sequences used for MAP7-MTBD and CKK constructs

**Primer name**	**Primer sequence**
MAP7_59fw	TGCCTGTGTTACGTGTTGATGAC
MAP7_59fwl	GCCGCGCGGCAGCCTGCCTGTGTTACGTGTTGATGAC
MAP7_170rv	TCAAGGGCTCCCATGGAGAGAGC
MAP7_170rvl	CAAGAAGAACCCCTCAAGGGCTCCCATGGAGAGAGC
CKK:CAM1-1474F_BamHI	CGGGGATCCGGTCCCAAGCTCTTTAAG
CKK:CAM1-1613R_SalI	ACGCGTCGACTCATTTACGAGTCTGGGCC
