# Supplementary material for: Direct observation of dynamic protein interactions involving human microtubules using solid-state NMR spectroscopy
Source: Nat Commun. 2020 Jan 2;11:18. doi: 10.1038/s41467-019-13876-x (PMC6940360; doi:10.1038/s41467-019-13876-x)
Supplement: Supplementary file 5 — Description of Additional Supplementary Files [file 41467_2019_13876_MOESM5_ESM.pdf]

Title: Supplementary Movie 1. Dual-color video of a polymerizing MT using HeLa S3 purified tubulin

Description: A low percentage of rhodamine-labeled porcine tubulin (3%) and 20 nM GFP-EB3 was used to visualize the MT lattice and ends respectively. MTs were grown from stable HiLyte-488-labeled GMPCPP MT seeds. Images were collected using a TIRF microscope at a 2-s interval. Video is sped up 90 times and time is shown in the format of minutes.
